# Perinatal Stem Cell Therapy to Treat Type 1 Diabetes Mellitus: A Never-Say-Die Story of Differentiation and Immunomodulation

**DOI:** 10.3390/ijms232314597

**Published:** 2022-11-23

**Authors:** Francesca Paris, Valeria Pizzuti, Pasquale Marrazzo, Andrea Pession, Francesco Alviano, Laura Bonsi

**Affiliations:** 1 Unit of Histology, Embryology and Applied Biology, Department of Experimental, Diagnostic and Specialty Medicine, University of Bologna, 40126 Bologna, Italy; 2 Pediatric Unit, IRCCS Azienda Ospedaliero-Universitaria di Bologna, 40138 Bologna, Italy

**Keywords:** diabetes, amniotic membrane, umbilical cord, perinatal cells, placenta stem cells, amniotic epithelial cells, Wharton’s jelly, cell therapy, regenerative medicine

## Abstract

Human term placenta and other postpartum-derived biological tissues are promising sources of perinatal cells with unique stem cell properties. Among the massive current research on stem cells, one medical focus on easily available stem cells is to exploit them in the design of immunotherapy protocols, in particular for the treatment of chronic non-curable human diseases. Type 1 diabetes is characterized by autoimmune destruction of pancreatic beta cells and perinatal cells can be harnessed both to generate insulin-producing cells for beta cell replenishment and to regulate autoimmune mechanisms via immunomodulation capacity. In this study, the strong points of cells derived from amniotic epithelial cells and from umbilical cord matrix are outlined and their potential for supporting cell therapy development. From a basic research and expert stem cell point of view, the aim of this review is to summarize information regarding the regenerative medicine field, as well as describe the state of the art on possible cell therapy approaches for diabetes.

## 1. Introduction

Diabetes mellitus (DM) is a complex metabolic disease characterized by blood hyperglycemia, attributable to a deficiency in insulin production or to a reduction in insulin uptake by peripheral cells. DM is a highly heterogeneous disease, but most patients can be classified under three conditions: “type 1 diabetes mellitus” (T1DM), which results in absolute insulin deficiency; “type 2 diabetes mellitus” (T2DM) is characterized by insulin resistance on the part of the peripheral cells; and “gestational diabetes” is defined as insulin intolerance that occurs during pregnancy [1,2].

Pancreatic beta cells synthesize, store, and uniquely release insulin in the human body [1]. This review will be mainly focused on T1DM, which is characterized by an immune-mediated destruction of pancreatic beta cells that leads to complete insulin deficiency [3]. T1DM onset can be at any age, but often is diagnosed in children around 5–7 years old, there being approximately 490,000 children affected worldwide [4,5]. Even if much progress has been made in terms of therapies in the last few years, currently, subcutaneous daily administration of exogenous insulin is the most significant treatment for T1DM [3]. The only long-term treatments available are pancreatic or islet transplantation from cadaver donors [6]. The shortage of organ donors and the need to combine chronic immune-suppressive treatments are significant issues [7]. Side effects related to long-term insulin injection are by no means negligible, which is why there is great interest in the use of stem cells (SCs) for the regeneration of pancreatic tissues [8,9,10].

The purpose of this review is to summarize the current knowledge about the use of perinatal cells for the treatment of T1DM, with a focus on their differentiation potential and immunomodulatory capability. The ideal SCs for regenerative medicine applications are pluripotent stem cells (PSCs) due to their ability to differentiate into any cells of the three germ layers [11]. The currently available PSCs that could be employed in clinical practice are embryonic stem cells (ESCs), induced pluripotent stem cells (iPSCs), adult stem cells (ASCs), and perinatal cells. ESCs and iPSCs have the capacity to differentiate and regenerate the beta cells islet, which helps to overcome the massive loss of beta cells during T1DM [12,13,14]. However, isolation of ESCs requires destruction of the blastocyst and for this reason use of them has met with ethical controversy. ESCs also display a unique combination of histocompatibility genes, which involves finding matching donors. Moreover, both ESCs and iPSCs display an intrinsic propensity for teratogenic and tumor formations [15]. ASCs are rare PSCs isolated from adult tissues, and unlike other SC populations, ASCs do not entail ethical controversy and risk of teratomas formations, but are quite rare, and usually, the recruitment of adult tissues is an invasive and uncomfortable procedure [16].

## 2. Perinatal Cells

In recent years, particular interest has been given to perinatal cells and their potential application in regenerative medicine [17,18]. Perinatal cells are considered an intermediate stage between ESCs and ASCs and display several advantages, including ease of isolation and the high yield in terms of cell number, as well as the possibility of expanding them in vitro for several culture passages [19]. Moreover, since placenta and the fetal annexa that comprise amniotic fluids, umbilical cord and fetal membranes are discarded after birth, and the resulting cells are free of ethical implications [20,21]. From perinatal tissues, it is possible to isolate several cell types, and for this reason, an extensive nomenclature for all perinatal cells was drawn up by Silini et al. [19]. Considering the complex gamut of the perinatal tissues, this review is focused in particular on amniotic epithelial cells (AECs) and mesenchymal stem/stromal cells (MSCs) isolated from amniotic membrane, Wharton’s jelly (WJ) and amniotic fluid [22].

Amniotic membrane derives from the inner cell mass of blastocysts; it is an avascular membrane and consists of five layers: the epithelium cells layer, the basement membrane layer, the compact layer, the fibroblast layer, and the spongy layer [23,24]. AECs are isolated from the epithelium layer and arise from the pluripotent epiblast about 8 days after fertilization, such that a certain proportion of AECs in term placenta retain the plasticity and pluripotency features typical of ESCs. On the other hand, amniotic membrane mesenchymal stem cells (AM-MSCs) are localized in the fibroblast layer of the amniotic membrane. Unlike AECs, which derive from the embryonic ectoderm, amniotic mesenchymal cells derive from embryonic mesoderm [25].

The umbilical cord is a fetal annexa characterized by the presence of two arteries and a vein buried within WJ, which is a gelatinous connective tissue mainly composed of an extracellular matrix rich in mucopolysaccharides, particularly hyaluronic acid and collagen fibers [26]. In 1991, McElreavey et al. first described the presence of MSCs in WJ [27]. These perinatal cells have demonstrated the capacity to respond to differentiative stimuli, in terms of osteogenic, chondrogenic, adipogenic, and also endocrine-pancreatic differentiation stimuli [21]. Moreover, it is known that these cells interact with the immune system by inhibiting activation and meanwhile stimulating the tolerogenic cell component [28]. The immunomodulatory potential of the perinatal cells population could constitute a valid approach for limiting the hyper activation of the immune system against beta pancreatic tissues, and therefore, modulating the inflammatory response in T1DM.

### 2.1. Amniotic Epithelial Cells (AECs)

The epithelial cells of the amniotic membrane are cells with cuboidal morphology, and constitute a monolayer in the innermost zone of the amniotic membrane, in direct contact with the amniotic fluid. The amniotic membrane is an easily accessible cell source; hence, AECs can be isolated with high yield and viability [29,30].

Despite variability among donors and protocols, the trypsin/Ethylenediaminetetraacetic acid (EDTA) digestion of the amniotic membrane is a widely used method for AEC isolation [31]. Briefly, incubation with trypsin/EDTA promotes the dissociation of cells from the basal membrane of the amniotic membrane, and harvested cells are then collected and seeded in the proper culture medium, which requires the addition of epithelial growth factor (EGF) [32] and expands for up to five passages [33].

The expression of PSC markers including NANOG, OCT-4, SSEA-3, SSEA-4, TRA1-60, and TRA1-80 in AEC population has been reported by several studies [34]. Although in the early stages of placenta development, most AECs express pluripotency markers, the heterogeneity of this perinatal cell population increases during pregnancy. Thus, the amniotic membrane at term is covered with a cells with heterogeneous degree of stemness and only a small percentage of AECs maintain PSC features [34,35]. AECs are positive for cytokeratins and express typical mesenchymal markers CD44, CD73, CD90, and CD105, while CD31, CD34, and CD45 are negligibly expressed [19,36].

#### 2.1.1. Differentiating Potential of AECs

Due to their early development, AECs retain characteristics found in PSC populations.

AECs have been successfully induced to differentiate towards all three germ layers under specific culture conditions [37,38], displaying an extremely high level of differentiation plasticity following chemical induction, biological treatment, and gene transfection, both in mono- and co-culture conditions [39].

The differentiation of AECs into endoderm lineage, in particular functional hepatocytes, was successfully demonstrated by Marongiu and co-workers [40], who showed the expression of typical liver markers both in vitro and in vivo. Moreover, corneal epithelial-like cells [41], and neuronal-like population were obtained after specific induction of AECs [42].

Importantly, several studies have focused on the ability of AECs to differentiate into the pancreatic-endodermic lineage, an increasing number of protocols have been developed over the years [43]. As early as 2003, a study reported that the use of nicotinamide as single inducing agent in AECs in vivo, normalized the blood glucose level after several weeks of implantation into streptozotocin (STZ)-induced diabetic mice, while anti-insulin anti-body staining was positive in the mouse tissue [44]. Later, Hou et al. [45] showed that AECs cultured in nicotinamide-supplemented medium were able to produce insulin, exhibiting glucose responsiveness by the release of c-peptide. However, the nicotinamide-induced differentiation process did not efficiently recapitulate the beta cell development pathway, nor did the differentiation protocol lead to a high yield of insulin and PDX-1 immuno-positive cells [45].

The tuning of stepwise protocol mimicking the pancreatic development from the definitive endoderm until maturation into pancreatic endocrine cells, using specific induction cocktails, has improved the efficiency of in vitro differentiation strategies [46]. However, the therapeutic application and long-term efficacy of currently available protocols are limited, due to the low efficiency of differentiation strategies and the generation of poly-hormonal populations [47].

Recently, the combination of hyaluronic acid with commonly used inducing factors was associated with an improvement in AEC differentiation into insulin-producing cells (IPCs). Moreover, hyaluronic acid supplementation contributed to the regulation of the immune and inflammatory response led by AECs, and to the restoration of damaged islet structures in T1DM diabetic mice [48].

The need for in vitro models mimicking the in vivo environment with more accuracy has led to development of three-dimensional (3D) culture strategies. Compared to the traditional two-dimensional (2D) culture, 3D models better represent the interactions occurring in vivo among different cell populations and the surrounding micro-environment, thus providing more accurate data about cell communication, drug metabolism, as well as cell–cell and cell–ECM interactions. Moreover, SCs cultured in 3D structures showed an improvement in their differentiation potential and viability, as well as in the paracrine secretion of cytokines, while increasing their anti-apoptotic and anti-oxidative capacities [49]. For these reasons, an increasing number of differentiation protocols have been developed using 3D-cultured SC populations.

Our group induced AECs to form 3D spheroids and to differentiate them into IPCs by culturing on an extracellular matrix and in serum-free conditions. After a stepwise differentiation protocol, a glucose-dependent insulin secretion and the release of c-peptide were detected in the differentiated spheroids [21].

Lebreton and co-workers set up organoids of dissociated islet cells (ICs) and AECs. Their results showed that the incorporation of AECs increased the synthesis and release of insulin in response to glucose, while enhancing engraftment, viability, and graft function after transplantation in animal models [50]. AECs have also been used as a support for transplantation of pancreatic islets in murine diabetes models, exploiting their anti-fibrotic and anti-inflammatory properties to improve islet engraftment and revascularization, reducing the risk of hypoxic damage [50,51]. Recently, Bosotti and colleagues generated organoids made of rat ICs with the addition of AECs and human umbilical vein endothelial cells (HUVECs). The addition of these two cell populations improved islet function in vitro, while an improvement in engraftment and neo-vascularization was observed after transplantation into a murine model [52].

#### 2.1.2. Immunomodulatory Capacity of AECs

Cell populations deriving from placenta are involved in the achievement of feto-maternal tolerance, which naturally avoids the immune mediated rejection of the embryo during pregnancy. The modulation of the immune response is achieved both by cell–cell contact and by the release of soluble signals [53].

The immune-privileged phenotype of AECs has been observed in vitro [54] and in vivo, following allogenic transplantation in animal models [55,56]. The tolerogenic profile of AECs is due to a low expression of human leukocyte antigen (HLA) class Ia molecules (HLA-A, -B and -C) and due to the absence of both HLA class II (HLA-DR) and costimulatory molecules [57].

On the contrary, AECs express the non-canonical HLA class Ib molecules (HLA-G, -E, -F) [58], which modulate the immune response under both physiological and pathological conditions [59]. Among HLA Ib molecules, HLA-G has been the most characterized, and its role in maintaining maternal-fetal tolerance has been widely demonstrated [60].

The HLA-G is highly expressed in isolated AEC and its expression is maintained during in vitro expansion, as well as in cryopreserved cells [58]. The immune-suppressive action of HLA-G is due to both membrane (mHLA-G) and soluble isoforms (sHLA-G), which modulate the response of several immune cell populations.

AECs can inhibit the cytotoxicity of natural killer (NK) cells [61] and reduce the activation of dendritic cells (DC) and B cells [62]. As regards T lymphocytes, AECs markedly suppress their activation and proliferation in response to allogenic stimuli while inducing a polarization towards regulatory T cells (Treg: CD4+CD25+FoxP3+ T lymphocytes), along with a pronounced down-regulation of T helper type 1 (Th1) and T helper type 17 (Th17) cell subsets [63].

Moreover, as shown by Li et al. 2005, the Fas/FasL pathway may be partially involved in the apoptosis of B and T cell populations induced by isolated AECs, while the expression of migration inhibitor factor (MIF) counteracts neutrophil and macrophage migration [62].

AECs are also involved in regulating the inflammatory response; as previously shown, these cells secrete several anti-inflammatory factors, including interleukin-10 (IL-10) and prostaglandin E2 (PGE2). The AEC secretome counteracts the action of proinflammatory cytokines, such as tumor necrosis factor-alpha (TNF-α), interleukin-6 (IL-6), interleukin-8 (IL-8), and interferon-gamma (IFN-γ), and reduces the expression of matrix metalloproteinase [64]. AECs also prove able to reduce local fibrosis and collagen deposition in animal models of lung and hepatic fibrosis, by inhibition of transforming growth factor-beta (TGF-β) signal [65,66].

Moreover, the paracrine features of AECs increase their phagocytic activity and promote the anti-inflammatory phenotype (M2) of the macrophage population. As for the pro-angiogenic potential of this cell population, it has already been previously demonstrated [50,67].

All in all, the immune-privileged and immune-suppressive phenotype of AECs along with their anti-inflammatory properties supports their use in several immune-based and inflammatory disorders as well as in regenerative medicine strategies, for example lung [68] and liver fibrosis [69], ocular repair [70], spinal cord injury [71], and T1DM treatments.

Regarding T1DM, pancreatic islet transplantation is an established procedure for beta cell replacement therapy and is required for the normalization of glycemic control. However, the application of this procedure is cramped by the insufficiency of islet donors for transplantation and the risk of immune response and transplant rejection. To overcome such limitations, and avoid recourse to an immune-suppressive regimen in transplanted patients, several works have suggested the possibility of using SCs properties to reduce the risk of transplant rejection and a local inflammatory reaction, by modulating the pancreatic micro-environment [72].

The generation of hybrid structures, consisting of pancreatic ICs and AECs, has proven to be a highly effective method in countering immune response and rejection after islet transplantation. Compared to MSCs, AECs are more prone to form stable hybrids spheroids when co-cultured with ICs [50,73].

Islet/AEC cultures have shown a strong anti-proliferative effect on phytohaemagglutinin (PHA)-stimulated peripheral blood lymphocytes (PBLs), compared to islets without AEC addition. The inhibition of PBL proliferation has also been carried out in PBL cultured with AEC-conditioned medium [74].

Zafar et al. [75] showed that co-culture models of AECs and porcine islets reduced CD4+ proliferation in vitro and delayed the islet rejection after transplantation into immuno-competent mice, compared to islets alone. The AEC-islets construct was associated with an improvement to insulin-secretory capacity in response to glucose. Moreover, AECs protected ICs from hypoxic damage, preserving their viability and functionality. The immunomodulatory properties of AECs were also maintained in 3D culture under defined serum-free conditions [21].

The transplantation of islet pancreatic cluster–AEC constructs into STZ-induced diabetic severe combined immune deficiency (SCID) mice improved islet engraftment and beta cell mass, leading to a normalization of blood glucose level [50,51]. Additionally, the presence of AECs promotes angiogenesis [76] and enhances re-vascularization of the transplanted islet [50].

Recently, Lebreton and colleagues demonstrated that the culturing AECs with inflammatory cytokines IFN-γ, TNF-α and IL-1β increased their anti-inflammatory and immunomodulatory capacity and enhanced their cytoprotective potential on pancreatic islets in co-culture models [77].

### 2.2. Wharton’s Jelly Mesenchymal Stem/Stromal Cells (WJ-MSCs)

Over the last decade, most of the studies attempting the origination of (IPCs) used ESCs or iPSCs rather than MSCs. WJ-MSCs are umbilical cord derived cells that display properties similar to bone marrow-MSCs (BM-MSCs) and some similar to ESCs [78]. Even though these cells originate from the embryonic epiblast, they are non-controversial, as they are collected when the umbilical cord is discarded at birth [79]. Interestingly, the large world-wide number of newborns may result in the availability of WJ-MSCs. The storage of the umbilical cord for MSC isolation meets a broad ethical consent and availability. This perinatal tissue is already known as a source of hemopoietic SCs derived from cord blood, which have achieved considerable clinical relevance and tissue bank feasibility. WJ-MSCs retain a combination of most of the ESC and MSC markers in primary culture [79]. WJ-MSCs are described as having low immunogenicity and being easy to transfected with foreign genes [80]. Like other sources of MSCs, WJ-MSCs possess immune properties that are tolerated in allogeneic transplantation, because they express mRNA for HLA-G while they do not express the co-stimulatory surface antigens CD40, CD80, and CD86 [81]. WJ-MSCs can suppress the proliferation of stimulated lymphocytes, a mechanism known as immune suppression [81]. Like other perinatal cells, WJ-MSCs are considered more primitive than their adult counterparts and several reports in the last decade have advocated them for their superior immune-suppressive capacity [82] and tolerogenic profile [83].

Since 2014, WJ-MSCs has been tipped to become the new gold standard for MSC-based therapies [82]. There has been much consent towards Wharton’s jelly as an interesting source of cells for clinical application, and relevant for translation to the field of DM cell therapy [84,85]. The most recent significance of the WJ-MSC’s clinical effectiveness derived from clinical trials involving in severe inflammation-based diseases, such as severe acute respiratory distress syndrome (ARDS) [86,87,88] in the coronavirus disease 2019 (COVID-19) context [89,90,91]. However, although the safety and efficacy of MSCs in the treatment of T1DM have been validated in animals, they are currently in the small-sample clinical trial phase [92,93]. In 2013, WJ-MSCs were expected to be an effective strategy for treatment of T1DM, since the delivery of WJ-MSCs for the treatment of new-onset T1DM did not report any side effects [93]. The results of a meta-analysis published in 2021 ended with the suggestion that transplanting MSCs may have beneficial effects especially on T1DM [94]. Clinical trials were undertaken to investigate the effect of WJ-MSCs in patients with T1DM. A study by Lu et al. aimed to examine efficacy and safety after one repeated transplantation of allogeneic WJ-MSCs. The change of c-peptide was significantly higher in the MSC-treated group than in the control group in adult-onset, but not different between groups among juvenile-onset T1DM [95]. Very recently, it has been shown that when a suspension spray including WJ-MSCs is applied on the wound area, it accelerates healing in diabetic wounds [96].

#### 2.2.1. Differentiating Potential of WJ-MSCs

Administration of MSCs has been proposed as useful in attenuating the autoimmune processes which lead to the destruction of beta cells. The endocrine pancreatic differentiation of WJ-MSCs is likewise being intensively researched. In protease-based processing of Wharton’s jelly, needed to isolate MSCs, an over-digestion of tissue may result in diminished cellular viability. La Rocca et al. promoted an isolation protocol [83] for WJ-MSCs to avoid diminished cellular viability and altered cellular function. They showed that non-enzymatically isolated WJ-MSCs express CD73, CD90 and CD105 as core markers, while expressing early endodermal markers (such as GATA-4, -5, -6). In 2015, El-Demerdash et al. [97] suggested that, as compared with umbilical cord blood (UCB)-MSCs, WJ-MSCs were preferable for their higher in vitro differentiation into insulin-producing cells and ameliorated hyperglycemia in vivo in diabetic rats. A similar outcome emerged from a meta-analysis of clinical trials in patients with diabetes [98]. This study encouraged the banking of WJ-MSCs in addition to UCB-MSCs, which have less efficacy in both T1DM and T2DM, as is evident from the glycemic control status (serum levels of HbA1c), measured c-peptide levels and monitored daily insulin doses. In a study published in 2015, undifferentiated WJ-MSCs were injected into non-obese diabetic (NOD) mice. After cell injection, there was the appearance of islet-like cell clusters in pancreas of transplanted mice. The outcome was an increase in animal survival, human c-peptide, and serum insulin levels and a decrease in glucose blood levels. Moreover, Treg cell number increased, while auto-aggressive T-cells and inflammatory cytokines levels decreased [99]. A protocol for in vitro neogenesis of pancreatic islet beta cells releasing insulin and c-peptide starting from non-human (chicken) MSCs [80] demonstrated the importance of Retinoic Acid (RA) and Nicotinamide in inducing critical signals for pancreatic beta cell development and maturation; they formed part of a cocktail of differentiating agents that also contained 5-Aza and activin A. Kassem et al. [100] revealed an association between the level of pluripotency markers in WJ-MSCS and the beta cell differentiation outcome, claiming that sustained expression of Oct-4 and Sox-2 pluripotency markers discourages WJ-MSC mature differentiation into IPCs. On investigating the role played by exendin-4 in the beta cell differentiation process, they found that the protocol incorporating this molecule induced the expression of Pdx-1 and Nkx2.2 beta cell markers, thus improving the differentiation outcome of WJ-MSCs into IPCs [101]. Further confirmation of Pdx-1 gene expression was reported by Ranjbaran et al. [102], where ELISA results showed that induced cells with activin A, RA, nestin, forskolin, and fibroblast growth factor-10 secreted more insulin than the uninduced WJ-MSC group. A lower differentiation potential was shown in WJ-MSCs than with menstrual blood-derived MSCs, following use of a three-step pancreatic differentiation protocol, which included RA (first); nicotinamide, EGF, and bFGF (second); and exendin-4 (third) [103]. Pre-treatment or over-expression of specific factors has been proposed as beneficial for WJ-MSC beta cell-differentiation. For example, apelin, which is involved in insulin sensitivity, has a short half-life that was prolonged by lentiviral expression in human WJ-MSCs. Transplantation improved glucose disposal and promoted endogenous pancreatic beta cell proliferation in vivo in a high-fat feed and STZ-treated rat model [104]. Apelin-WJ-MSCs increased insulin and c-peptide levels while decreasing TNF-alpha in the plasma. Modification by epigenetic regulators can be a strategy to achieve better in vitro IPCs starting from WJ-MSCs.

When two histone deacetylase (HDAC) inhibitors were tested, the pre-treatment with TMP269 improved the expression of insulin, somatostatin, GLUT2, PDX-1, PAX4, and NKX6.1 pancreatic markers [96]. Beikmohammadi et al. [105] transfected WJ-MSCs in order to generate hepatocyte-like cells and differentiated them by using serial exposure to different inducing material and exogenous growth factors. In vitro, the cells released insulin in response to high glucose levels. Differentiated IPCs can significantly improve blood glucose levels in diabetic rats due to the continuous secretion of insulin by transplanted cells which survive in the diabetic rat islets. Transplantation of undifferentiated human WJ-MSCs can significantly improve insulitis and re-balance the inflammatory condition in diabetic rats with only a slight decrease in blood glucose levels. Hsiao et al. compared the therapeutic effect between differentiated (multi-step with activin-A, taurine, glucagon-like peptide 1 (GLP-1), and many other factors) and undifferentiated human WJ-MSC in STZ-treated mice [106]. The results showed that differentiated cells improved blood glucose levels due to insulin secreted by transplanted cells surviving in islets, while undifferentiated cells improved the inflammation milieu, but had only a slight effect on glucose levels. Recently, the combination of melatonin with WJ-MSCs was investigated in diabetic mice and the pre-treatment with melatonin alleviated blood glucose levels [107]. In another study, some decellularized matrices were used as a scaffold to support the differentiation of WJ-MSCs into IPCs. A decellularized umbilical cord scaffold enhanced the expression of GLUT-2 and PDX-1 in the IPCs, while insulin gene expression and protein secretion were enhanced in comparison with cells non-cultured on decellularized matrix [108].

#### 2.2.2. Immunomodulatory Capacity of WJ-MSCs

MSC homing onto the target site depends on the composition of the local microenvironment; MSCs can exhibit therapeutic responsive polarization into either anti-inflammatory or pro-inflammatory phenotypes [109]. In vitro activation of specific toll-like receptors (TLRs) in MSCs has a profound effect on the MSC immunomodulatory phenotype. Thus, ex vivo priming of MSCs via specific molecules mimicking pathogen-associated molecular patterns (PAMPs) could be a useful tool in cell therapy prior to their infusion in vivo [110]. For example, lipopolysaccharides (LPS) preconditioning of WJ-MSCs generates extracellular vesicles (EVs) that alleviate inflammation and enhance wound healing in STZ [111]. In general, it is thought that MSC priming by pro-inflammatory modes stimulates their anti-inflammatory phenotype, potentially augmenting its anti-inflammatory potential in vivo [112].

MSCs possess the ability to assemble in 3D structures, as a reminiscence of their aggregation in mesenchymal condensation during embryogenesis [112]. By providing spatial organization and by increasing intercellular interactions, 3D cultures of MSCs can enable a closer recapitulation of the in vivo MSC niche. A decade ago, a report suggested that culturing as 3D aggregates could increase the therapeutic potential of MSCs and, by secreting substantial quantities of anti-inflammatory proteins, prove more effective than 2D culture in therapies for diseases characterized by tissue injury and unresolved inflammation [111]. In fact, MSC spheroids, from data collectively available from bone marrow- derived cells, show an enhanced anti-inflammatory effect associated with high expression of TGF-β1, IL-6, TSG-6, and PGE-2 [110]. The 3D aggregation method, the size of the aggregate, and the kinetics are culture parameters that influence the paracrine immunomodulatory function, so that controlling MSC aggregates may be an avenue that leads to optimizing the immunomodulatory function [113].

In 2016, Al Madhoun et al. sketched a method of developing differentiation towards the pancreatic lineage utilizing 3D spheroids; they defined a serum-free protocol designed to differentiate WJ-MSCs into definitive endoderm cells. After 7 days, they found CXCR4 protein expression in 85% of cells in addition to gene expression of Sox17 and FoxA2 markers [114]. A higher yield of EVs exhibiting enhanced immunomodulatory properties was observed with dynamic 3D aggregate culturing of WJ-MSCs, which resulted in the reduction of proliferating CD8+T cells, reduced cellular senescence and reactive oxygen species (ROS), and accelerated wound closure in a scratch assay [115]. Following glucose challenge, Seyedi et al. found that suspension culturing (e.g., hanging drop cultures) resulted in a higher expression of insulin protein and in vitro secretion than did the conventional culture method commonly used in IPC [116]. Three-dimensional ultra-low attachment (ULA)-based culturing of WJ-MSCs enhanced protein expression of the triad of pluripotency transcription factor markers when compared to 2D-cultured cells [115]. Importantly, the same recent study demonstrated that the genes of certain immunomodulatory factors, Indoleamine 2, 3-dioxygenase (IDO), IL-10, and leukemia inhibitory factor (LIF), but also other proteins such as angiopoietin-1 (ANG1) and vascular-EFG, were significantly up-regulated in ULA-3D cultured WJ-MSCs. Moreover, definitive endodermal markers were also up-regulated, in particular CXCR4 at an mRNA level and SOX17 and FOXA2 at a protein level. Three-dimensional culture systems have been constructed using different substrates (collagen, chitosan, or polylactic-co-glycolic acid) to evaluate the immunomodulatory characteristics in WJ-MSCs [116]. Compared to the 2D condition, 3D cultures yielded higher stemness, while a pathway such as the TLR signal proved to be enriched. Concerning the immunophenotypic pattern, 3D cells retain low immunogenicity properties (CD80, CD86, HLA-DR), despite the down-regulation of CD73 [116]. Importantly, 3D culturing enhanced the immune-suppressive function, as seen in T lymphocyte proliferation. This study interestingly showed how 3D geometry can influence the immunomodulatory properties of MSCs, and indicated that MSCs might play a stronger immune-suppressive role in vivo, again suggesting that MSC transplantation as suitable in clinical practice.

#### 2.2.3. Co-Culture Strategies Based on WJ-MSCs

Many studies have shown that co-culturing based on MSCs is beneficial for in vitro islet generation, and for allogenic islet transplantation. WJ-MSCs could play a dual role in beta cell generation, acting as a pro-differentiation agent and protecting differentiated beta cells. Thus, clusters formed of WJ-MSCs, following a protocol adapted from Chandravanshi et al. [117], involving supplementation of GLP-1, nicotinamide, and taurine, express pancreatic markers (c-peptide, glucagon glut2, and insulin), lose expression of vimentin and CD90 in comparison with control WJ-MSCs, and secrete insulin with glucose stimulation [118]. Compared to insulin producing clusters without MSCs, co-cultures with WJ-MSCs amplified TGFβ anti-inflammatory cytokine, vascular-EGF, and platelet-derived growth factor subunit A (PDGFA) angiogenic cytokines, as well as reducing pro-inflammatory cytokines (e.g., TNFα and IL1β). In addition, WJ-MSC co-cultures with differentiated clusters limited oxidative stress while protecting from normoxia damages and hypoxia-induced apoptosis. Oxygen levels seem to have a slight influence on the outcome of MSC transplantation as treatment for diabetes, as suggested by a study where co-transplantation of hypoxia pre-cultured MSCs and islets accelerated glycemic utilization in a mouse model [119]. Murine islets co-cultured with human WJ-MSCs were protected from dysfunction caused by hypoxia, one of the major factors impairing islet graft function [120]. In another study, conditioned medium (CM) of WJ-MSCs protected neonatal porcine ICs clusters exposed to hypoxia, and WJ-MSCs exosomes released in CM increased islet insulin secretion in a concentration-dependent manner [121]. Additionally, the insulin and c-peptide levels increased in rat pancreatic cells co-cultured with human WJ-MSCs while the blood glucose levels diminished in diabetic rats [122]. Recently, the levels of several cytokines in the supernatant of human islets cultured alone were compared to islets in direct or indirect co-culture with WJ-MSCs [123]. The study showed a significantly higher secretion of both anti-inflammatory and inflammatory cytokines in direct co-cultures than in mono-cultured islets or in indirect co–cultures. Other tissue models were indirectly used for evaluating the effect of CMs of WJ-MSCs, which improved insulin resistance by multiple mechanisms in C2C12 cells [124].

Considering the evidence, in vivo co-administration seems to be another option in stem cell therapy for diabetes. In confirmation of this, a pilot randomized controlled open-label clinical study conducted by Cai et al. evaluated the effect of a co-transplantation of 1.1 × 10^6^/kg WJ-MSC and 106.8 × 10^6^/kg bone marrow mononuclear cells through supra-selective pancreatic artery cannulation in T1DM patients. One year after treatment an increase was reported in the c-peptide area under the curve (AUC) and insulin AUC, and a reduction in hemoglobin A1c (HbA1c), fasting glycemia, and daily insulin requirements in the treated group [92]. Moreover, in a very recent study published in 2022, Wu et al. analyzed the incidence of diabetic co-morbidity in an eight-year follow-up of the previous clinical trial. In the treated group, a significant reduction was observed in the incidence of chronic diabetes complications such as neuropathy, diabetic nephropathy, and retinopathy [124].

### 2.3. Other Sources of Perinatal Cells

#### 2.3.1. Amniotic Fluid Stem Cells (AFSCs)

Amniotic fluid stem cells (AFSCs) are usually collected during the amniocentesis procedure. Human AFSCs isolated during the second and third trimester express many pluripotent marker such as SSEA3, SSEA4, NANOG, and c-MYC [125]. AFSCs also display high expression levels of characteristics MSC markers, CD29, CD44, CD73, CD90, and CD105 [125]. Similarly to AECs and WJ-MSCs, AFSCs are not tumorigenic and present both immunomodulation and differentiation capacity [126,127,128]. AFSCs can differentiate along adipogenic, osteogenic, myogenic, endothelial, neurogenic, and hepatic pathways [126]. Moreover, Mu and co-workers [129] fully differentiated AFSCs into IPCs though a multi-step protocol. The differentiated cells expressed insulin, Glut2, Nkx6.1, and glucokinase.

Administration of human AFSCs in a diabetic mouse model brought a reduction in the time of wound healing and a reduction of local inflammation, because of the increased secretion of IL-6, which is responsible for polarizing of macrophages in a pro-repair status [129]. Likewise, Sato, Y. et al. [130] reported the ability of AFSCs to switch macrophages M1 to M2 in an animal model of perinatal sepsis, reducing inflammation. AFSCs could also have a direct role in the treatment of T1DM; Villani and colleagues [131] have demonstrated that administration of AFSCs in an animal model of diabetes has a protective effect, promoting endogenous beta cell functionality and proliferation. However, the beneficial effect was observed if AFSCs were injected before severe hyperglycemia occurred.

#### 2.3.2. Amniotic Membrane Mesenchymal Stem Cells (AM-MSCs)

Amniotic membrane mesenchymal stem cells (AM-MSCs), like mesenchymal stem cells from classical sources, display a significant immunomodulation and differentiation capacity. AM-MSCs and the resulting CM can suppress both innate and adaptive immune responses through the inhibition of T cells activation and proliferation. AM-MSC-CM can also promote regulatory T cells, induce a down-regulation of Th1 and Th17 cell populations and reduce the cytotoxicity of NK cells [132,133]. Moreover, AM-MSCs can reduce the activation of DCs, blocking both differentiation and maturation of monocytes [134]. Magaña-Guerrero and co-workers [133] also investigated the ability of AM-MSCs to interact with neutrophils, reducing neutrophil extracellular trap (NETs) release and ROS production from LPS-stimulated mouse bone marrow-derived neutrophils.

Kharat et al. [135] demonstrated the capacity of AM-MSCs to differentiate into IPCs using a combination of insulin-like growth factor-1 (IGF-1) and somatocrinin. The differentiated cells were positive for c-peptide. Kadam et al. [136] also observed that AM-MSCs can assemble in islet-like clusters (ILC) exposed to specific growth factors and differentiating agents. Those differentiated ILCs showed expression of human insulin, glucagon, and somatostatin. The administration of differentiated AM-MSCs in experimental diabetic mice resulted in restoration of normoglycemia without immune rejection. These studies suggest that AM-MSCs could be a valid player in the treatment of T1DM due to both their differentiative and immunomodulating properties.

## 3. Discussion

**Figure 1:**
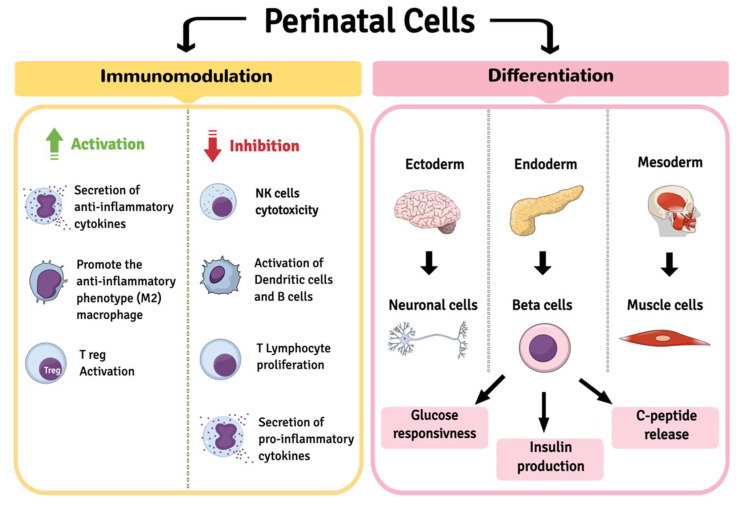
The double ability of perinatal cells in sustaining anti-diabetes cell therapy strategies. One activity of perinatal cells is to promote anti-inflammatory effects and to regulate pro-inflammatory response, both by using their own secretome and by interacting with different immune cells. In addition, the cell ability to differentiate, particularly into the endoderm lineage, makes perinatal cells ready to be exploited during directed or induced differentiation towards beta cell commitment, pre-transplantation or post-transplantation. The limiting activity on patient auto-immune response and the new production and release of insulin in vivo are expected to be one of the chief challenges in regenerative medicine for diabetes treatment.

In the field of cell therapy for T1DM, not only does cell regeneration represent an important curative strategy, but immunotherapy and its possible applications may also help patients suffering from this chronic disease in the future. Cell therapies represent a new tool of regenerative medicine; among the possible cell populations cells from perinatal tissues have marked features (Figure 1) that combine immunomodulation capacity and greater differentiation ability than MSCs derived from an adult source. These features are described in several studies, listed in Table 1.

The immunomodulatory properties of perinatal cells are evident and well described. Recently, their secretome has been reported to play an essential role, but not all bioactive factors have been identified [155]. These properties are shared by both the epithelial and the mesenchymal cells that constitute the perinatal tissues.

As regards T1DM, an increasing number of studies suggest that transplanted islets may benefit from the immunomodulatory and anti-inflammatory features of AECs [74]. The incorporation of this perinatal cell population into pancreatic islets modulates the immune response and promotes neo-angiogenesis, thus enhancing the engraftment and survival [156]. Many of the AEC functions are exerted by the release of soluble paracrine factors into the surrounding microenvironment [64,157]. The anti-inflammatory and anti-fibrotic properties of AECs are shared with mesenchymal cells deriving from perinatal tissues, particularly AM-MSCs and WJ-MSCs [158,159].

Several studies have highlighted the potential of WJ-MSC secretome for treating diabetes [99,160]. In this connection, one interesting future prospect would be harnessing the therapeutic effects of soluble secreted molecules, for example WJ-MSC-CM, as already pre-clinically reported. Utilization of the WJ-MSC secretome will be a cell-free tool for regenerative medicine applications. In parallel, factors responsible for MSC-driven immunomodulation can be entrapped in EVs. MSC-EVs appear warranted for the translational treatment of diabetic wounds [161]. Following the example and the study presented by Silini et al., which showed that CM from human amniotic membrane has comparable immunomodulatory properties to AM-MSCs-CM [162]. Future studies may investigate whether the umbilical cord itself can maintain an immunomodulatory capacity similar to WJ-MSCs. This would open up an important possibility of improving the scalability of the good manufacturing practice (GMP) production process while reducing efforts concerning MSC culturing. The need to continue studying the parameters that could impact on clinical application, for example unfractionated cell versus sorted cell potential, 11/18/22 11:01:00 AMhas opened a debate as to defining the optimum culture condition of MSCs [163]. In this review of the literature, we have highlighted the potential of AECs and WJ-MSCs, as well as other perinatal cells, for being interesting tools to develop in designing diabetes cell therapy. It appears that, despite reports of efforts to produce beta cells in vitro, currently no clinical transplantation of such differentiated cells has happened. In this situation, the best potential use of perinatal cells for diabetes treatment must surely reside in their immunomodulating efficacy.

In the last decade, several clinical trials involving the use of WJ-MSCs in T1DM patients have been proposed. Hu et al., in 2013, evaluated the long-term effects of WJ-MSC transplantation in newly onset T1DM patients. This study demonstrated that WJ-MSC administration can restore the function of islet beta cells, even though the exact mechanism is still not understood [93].

In 2020, a phase I–II clinical trial (ClinicalTrials.gov Identifier: NCT03406585) regarding the use of WJ-MSCs in T1DM patients, conducted by Per-Ola Carlsson, was concluded. It was a double-blind randomized trial with WJ-MSCs for preserving endogenous insulin production in adult patients diagnosed with T1DM. Despite the results of this study still not being available, an ongoing trial (ClinicalTrials.gov Identifier: NCT03973827) conducted by the same research group is focused on the effects of repeated WJ-MSC treatment in adult patients diagnosed with T1DM. Recently, another ongoing phase I–II clinical trial (ClinicalTrials.gov Identifier: NCT05061030) regarding the administration of WJ-MSCs in children and adolescents with T1DM started. The first results of this study are expected to be available in 2028.

It is a limitation of the present review that we did not study the potential of perinatal cells as a tool for gene therapy in diabetes, not only because molecular biology strategies lie outside our scope, but mainly because the native ability of SCs and their genetic stability are significant factors affecting the quality of MSCs; indeed, these intrinsic properties have made perinatal cells good candidates in various previous clinical settings. The physiological and minimal manipulated maintenance of perinatal cells will form the next goal of the thriving regenerative medicine field.

## 4. Conclusions

Common features of immunoregulation among perinatal cells and the search for culture parameters such as in differentiation protocols will make a deep difference to elucidating the right cell therapy when it comes to combating diabetes. In general, it is important to remember that other factors can affect clinical application, such as the method of culture, cell heterogeneity, and dosage, for example in MSC-based cell therapy for diabetes [164].

Selection and fractionation of MSCs or perinatal cells as well as the ratio building co-culture setting and 3D cultures (e.g., spheroids), are parameters urgently needing to be defined and characterized in vitro if we are to improve our chances of obtaining clinically effective SCs. In the future, the secretome of perinatal cells could contribute to the immune response against various inflammatory microenvironments, hoping the future diabetes treatment will involve this cell-free therapy approach. Many therapeutic agents supporting the function of ICs can have off-target effects and usually only a fraction reach the desired niche with limited access in the pancreas [164]. EVs and liposomes are appealing tools for realizing pancreas-targeted delivery, with the advantage of being bioengineered so as to transport selected cargoes.

More research is needed to decipher the immunomodulation mechanisms of perinatal cells and define their translational aspects. This will be of great benefit when it comes to designing the best clinical strategy tapping this natural medical resource and avoiding possible drawbacks for the T1DM patient.

## Figures and Tables

**Table 1:** The immunomodulatory capacity and differentiation potential of perinatal cells reviewed in this study.

Immunomodulation Activity	AESC	WJ-MSC	AFSC	AM-MSC
Inhibiting the cytotoxicity of natural killer cells	[137,138]	[139]	[133]	
Reducing of the activation of dendritic cells	[62]	[140]		[134]
Reducing B cell activation	[62]		[141]	
Anti-proliferative effect on activated PBMC	[21,74]	[142]	[128]	
	[143,144]	[142]		
Promoting the anti-inflammatory phenotype (M2) of macrophage populations	[145]	[146]	[129,130]	
Reducing T lymphocytes proliferation	[75,138]	[81,115,140]	[133]	[133]
In vivo allogenic transplantation	[37,51,75,147]	[84,85,99,112]		
Increased expression of: TGF-β1, IL-6, TSG-6, PGE-2	[148]	[112,140]		
Secretion of EVs	[115,149]	[115]		
Secretion of: IL-10, PGE2, hyaluronic acid		[112,140]		
Expression of HLA Ib	[57,58,59]	[81]		
Expression of migration inhibitor factor (MIF)	[62]			
**Differentiation capacity**	**AECs**	**WJ-MSCs**	**AFSCs**	**AM-MSCs**
Expression of pluripotency markers: NANOG, OCT-4, SSEA-3, SSEA-4, TRA1-60, TRA1-80	[34]	[150]	[151]	[152]
Pancreatic-endodermic lineage	[18,43,45]	[95,102,106,153]	[154]	[135,136]

Abbreviations: AEC: amniotic epithelial cells; WJ-MSC: Wharton’s jelly mesenchymal stem/stromal cells; AFSC: amniotic fluid stem cells; AM-MSC: amniotic membrane mesenchymal stem cells; PBMCs: peripheral blood mononuclear cells; Th1: T helper type 1; Th17: T helper type 17; TGF-β1: transforming growth factor beta 1; IL-6: interleukin 6; TSG-6: tumor necrosis factor stimulated gene-6; PGE-2: prostaglandin E2; EVs: extracellular vesicles; IL-10: interleukin 10; HLA-1b: Human Leukocyte Antigen 1b; MIF: migration inhibitor factor.

## Data Availability

Not applicable.
